# Liraglutide Improves Hypertension and Metabolic Perturbation in a Rat Model of Polycystic Ovarian Syndrome

**DOI:** 10.1371/journal.pone.0126119

**Published:** 2015-05-26

**Authors:** Vanessa Hoang, Jiangjiang Bi, Sheba M. Mohankumar, Arpita K. Vyas

**Affiliations:** 1 Department of Pediatrics and Human Development, Michigan State University, East Lansing, Michigan, United States of America; 2 Department of Veterinary Biosciences and Diagnostic Imaging, University of Georgia, Athens, Georgia, United States of America; 3 Department of Anesthesiology, Tongji Hospital, Huazhong University of Science and Technology, Wuhan, Hubei, China; University Medical Center Utrecht, NETHERLANDS

## Abstract

Polycystic ovarian syndrome (PCOS) is the most common endocrine disorder in women of reproductive age, with a prevalence of 5–8%. Type 2 diabetes and cardiovascular disease (CVD) are its long-term complications. Targeted therapies addressing both these complications together are lacking. Glucagon like peptide-1 (GLP-1) agonists that are used to treat type 2 diabetes mellitus have beneficial effects on the cardiovascular system. Hence we hypothesized that a GLP-1 agonist would improve both cardiovascular and metabolic outcomes in PCOS. To test this hypothesis, we used an established rat model of PCOS. Prepubertal female Sprague Dawley rats were sham-implanted or implanted s.c. with dihydrotestosterone (DHT) pellets (90 day release; 83μg/day). At 12 wks of age, sham implanted rats received saline injections and the DHT treated animals were administered either saline or liraglutide (0.2mg/kg s.c twice daily) for 4 weeks. Subgroups of rats were implanted with telemeters between 12-13 weeks of age to monitor blood pressure. DHT implanted rats had irregular estrus cycles and were significantly heavier than the control females at 12 weeks (mean± SEM 251.9±3.4 vs 216.8±3.4 respectively; p<0.05) and 4 weeks of treatment with liraglutide in DHT treated rats significantly decreased body weight (mean± SEM 294.75 ±3.2 in DHT+ saline vs 276.25±2.7 in DHT+ liraglutide group respectively; p<0.01). Liraglutide treatment in the DHT implanted rats significantly improved glucose excursion during oral glucose tolerance test (area under the curve: DHT+ saline 28674±310 vs 24990± 420 in DHT +liraglutide p <0.01). DHT rats were hypertensive and liraglutide treatment significantly improved mean arterial pressure. These results suggest that GLP-1 treatment could improve DHT–induced metabolic and blood pressure deficits associated with PCOS.

## Introduction

Polycystic ovarian syndrome (PCOS) is the most common endocrine disorder in women of reproductive age [1, 2]. The exact etiology of the condition remains unclear. Current prevalence is estimated at 5–8% [2]. The hallmarks of the syndrome are hyperandrogenism and ovulatory dysfunction. The syndrome is also closely associated with obesity, insulin resistance and type 2 diabetes mellitus[3]. Predisposition for type 2 diabetes, dyslipidemia, obesity and hypertension also place young women with PCOS at a higher risk for developing cardiovascular disease[4]. There is a growing body of evidence that suggests increased prevalence of cardiovascular disease in women with PCOS. Women with PCOS have 7-fold increased risk for myocardial infarction[5] and have a higher incidence of coronary artery disease [5–7] compared to healthy women. Moreover, hypertension has been shown to be prevalent in adolescent, peri- and post-menopausal PCOS patients [8–10]. Although these reports suggest that there is an association between cardiac dysfunction and PCOS [11] this needs to be studied further. This is because, current therapies either target the metabolic perturbation associated with the syndrome or the hyperandrogenism and ovulatory dysfunction that occur. With increasing incidence of cardiovascular dysfunction seen in this syndrome there is a need to focus on the cardiovascular health of these patients and implement new therapeutic strategies that not only resolve the reproductive and metabolic issues associated with the syndrome but also prevent/reverse cardiovascular dysfunction seen in PCOS patients.

Type 2 diabetes that is commonly associated with PCOS most likely occurs due to obesity and insulin resistance. Several conditions involving insulin resistance have been linked to an altered incretin effect. Incretin effect is a phenomenon whereby oral ingestion of glucose elicits a greater increase in insulin secretion than glucose administered via intravenous route [12] due to the release of gut hormones such as glucagon like peptide-1 (GLP-1) and gastric inhibitory polypeptide (GIP). GLP-1 is a 30-amino acid polypeptide that is secreted by the L cells in the intestine and has direct stimulatory effects on insulin secretion from the pancreas. In PCOS patients, there are significant changes in GLP-1 secretion. GLP-1 (incretin hormone) levels were found to be low during fasting and in the later phase of the oral glucose tolerance test (OGTT) and after a meal in PCOS women[13, 14]. It is likely that low GLP-1 levels may contribute to the metabolic dysfunction in PCOS. In fact, metformin, a widely used drug therapy for PCOS, increases the levels of GLP-1 [15]. Although low GLP-1 levels could contribute to the type 2 diabetes seen in PCOS, it is not clear if they could contribute to the cardiovascular deficits as well.

The GLP-1 receptor agonist liraglutide, is currently approved for use in type 2 diabetes to improve glycemic control. It also has beneficial effects on the cardiovascular system [16, 17]. Therefore, liraglutide could potentially improve both the metabolic and cardiovascular dysfunction observed in PCOS. The role of GLP-1 agonists in the treatment of PCOS is relatively unexplored. Recent studies have shown that a GLP-1 agonist may lower body weight in women with PCOS in conjunction with metformin[18]. Therefore, we wanted to investigate if treatment with liraglutide could reverse the cardiovascular and metabolic dysfunction seen in PCOS. For this purpose, we chose to use a well-established rodent model of PCOS created by exposing prepubertal rats to a pharmacological dose of dihydrotestosterone (DHT)[19]. The DHT-induced rat model of PCOS has been shown to develop reproductive and metabolic perturbation together with endothelial dysfunction and hypertension [19–23]. Therefore, it serves as an ideal model to study the effects of liraglutide treatment.

## Methods

### Model

Sprague Dawley rats were obtained (Charles River Laboratories, Wilmington, DE) at 4 weeks of age and housed in temperature controlled animal quarters with constant light/dark cycles (12 hours light/12 hours dark) with free access to standard chow and water. Between 4–5 weeks of age the rats were implanted s.c. with a non-aromatizable dihydrotestosterone pellet (DHT 7.5 mg/90 day with daily dose = 83 μg; Innovative Research America, Sarasota FL) at the back of the neck (n = 31) or had sham surgery with no pellet implant (n = 13). Starting at 8 weeks of age they had daily vaginal cytology and body weight measurements. At 12 weeks of age, a subgroup of the DHT-implanted female rats (n = 15) received twice daily s.c. injections of liraglutide 0.2mg/kg (Novo Nordisk, Plainsboro, NJ) and the rest received saline injections twice daily. Between 12–13 weeks of age, telemeters were implanted in a subgroup of the rats in the three groups, DHT+ liraglutide, DHT and controls, and telemetry data were recorded hourly and averaged every 12 hours. At around 16 weeks of age, a subgroup of rats had an oral glucose tolerance test in all three groups. Rats were euthanized by decapitation under isoflurane anesthesia between 88–90 days from DHT implantation during diestrus phase of the estrus cycles and serum was separated from trunk blood. Abdominal adipose tissue was collected and weighed. Serum was stored at -80°C until analysis. All protocols were reviewed and approved by the Institutional Animal Care and Use committee at Michigan State University (Approval number 03/13-050-00).

### Body Weight change

Rats were weighed at 4 weeks of age and then weekly from 9–13 weeks and again at 16 weeks of age.

### Vaginal Cytology

Animals were subjected to vaginal cytology on a daily basis starting from 8 weeks of age. A drop of the vaginal lavage was placed on a glass slide and air-dried. The smears were stained with Methylene blue. The cell composition of the smear was used to determine the stage of the estrous cycle as described previously [24].

### DHT levels

DHT levels were measured in duplicate using an ELISA kit obtained from My Biosource (San Diego, CA) at 88 days from DHT implantation using serum samples from all three groups. The assay was performed according to the manufacturer’s instructions.

## Metabolic Studies

### Glucose tolerance test

At 16 weeks of age, OGTT was conducted in a subgroup of rats (n = 9 control, n = 10 DHT treated and n = 8 DHT+ liraglutide) that were fasted for 10–12 hours overnight and received a 2 g/kg dose of dextrose by oral gavage. Blood glucose levels were measured using an Accu-check Aviva glucometer by tail cut bleed at 0, 30, 60, 90, 120 and 150 min post gavage.

### Insulin levels

Fasting serum insulin levels were measured at 16 weeks of age in all three groups of rats using rat insulin elisa kit (ALPCO, Salem, NH). The assay was performed per manufacturer’s guidelines.

### Total Cholesterol

Total cholesterol was measured in the serum between 88–90 days from DHT implant under fasted condition (fasted for 10 hours) using the Amplex kit (Molecular Probes, Life Technologies, Grand Island, NY) (n = 5 for control rats, n = 8 for DHT and DHT+ liraglutide).

### Telemetry

Telemeters were implanted in the rats at 12–13 weeks of age (n = 9–10 per group) under isoflurane anesthesia using aseptic technique as described previously [25]. Briefly, the femoral artery was visualized and a transmitter catheter was introduced through it with the tip residing in the abdominal aorta. The body of the transmitter was placed in a subcutaneous pocket in the abdomen. One week later, blood pressure data from the transmitter was collected continuously and data was analyzed over 15 days by averaging hourly recordings for day and night time separately. Data were stored and analyzed using the Dataquest A.R.T. software (Data Sciences International, St. Paul, Minnesota).

## Statistical Analyses

All data in the study are expressed as mean ± Standard error of the mean (SEM). Two way repeated measures ANOVA with post hoc Fisher’s LSD test was used for analysis of telemeter data, oral glucose tolerance test and body weights. One way ANOVA was used to determine differences in rate of body weight gain, serum cholesterol, DHT and abdominal fat. P value less than 0.05 was considered to be statistically significant.

## Results

### DHT levels and estrous cycles

**Fig 1:**
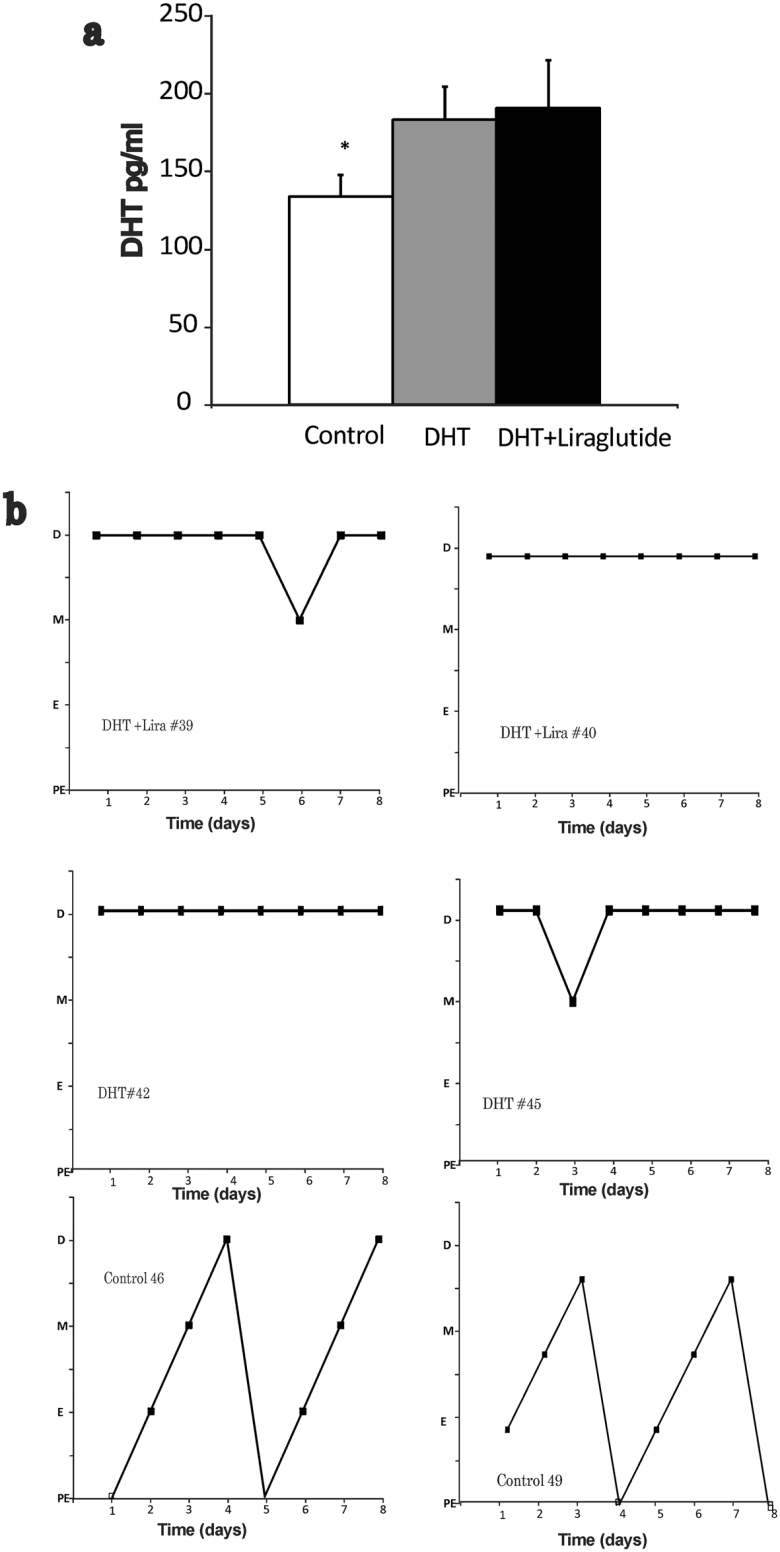
**DHT levels and estrous cycle pattern:** a) **DHT levels measured at 16 weeks of age.** (Mean± SEM; pg/ml)**:** measured in the serum by ELISA, control group (n = 9) DHT group (n = 10) and DHT+ liraglutide group (n = 11). DHT levels were 1.3 fold higher in the DHT treated rats compared to control irrespective of liraglutide treatment (p<0.05). b) **Estrous cycle pattern in the three groups.** Vaginal cytology was done every day starting at 8 week of life till the end of the study. Data presented are from the last week of the study. The figure shows pattern of two representative rats in each group including control, DHT and DHT+ Liraglutide. P: proestrus; E: estrus; M: metestrus; and D: diestrus.

As shown in Fig 1a, DHT levels were increased by 1.3 fold in the DHT-induced PCOS group and by 1.4 fold with liraglutide treatment compared to control. Vaginal cytology revealed that the DHT-induced PCOS female rats were acyclic and were in a state of persistent diestrous and this was unchanged with liraglutide treatment. Control rats were cycling normally (Fig 1b).

### Body weight

**Fig 2:**
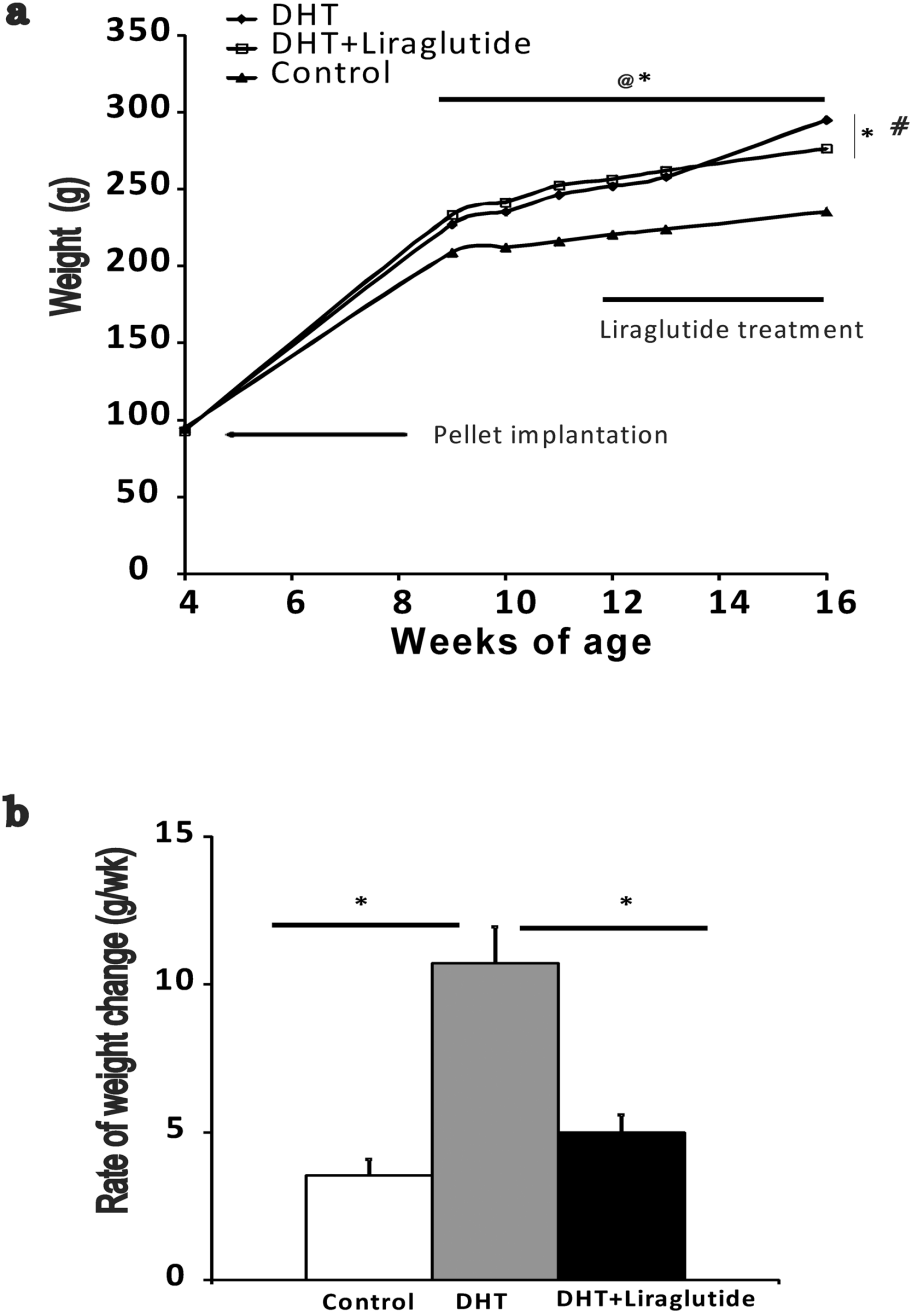
Effect of liraglutide on DHT induced weight gain. (Mean± SEM; g): a) Rats were weighed at 4 weeks and again from 9–13 weeks and at 16 week. “#” denotes p<0.05 between DHT and DHT+liraglutide and “@” denotes p <0.05 between DHT and control. b) Rate of weight change per week between 12–16 weeks. n = 12 rats in DHT and DHT+ liraglutide group n = 9 control group. * denotes p<0.05 between DHT +liraglutide and DHT and control.

As shown in Fig 2a, body weight of the DHT-induced PCOS females were significantly higher than the control females at 12 weeks (251.9±3.4 vs 216.8±3.4 respectively; p<0.05) and liraglutide treatment starting at 12 weeks of age significantly reduced body weight in the DHT treated rats by 16 weeks (294.75 ±3.2 in DHT vs 276.25±2.7 in DHT+ liraglutide group respectively; p<0.01). At this time point, body weight in the DHT+liraglutide group was still higher than that in control animals (230.7±5.4; p<0.05). The rate of body weight gain was significantly higher in the DHT-treated group compared to control. However, treatment with liraglutide significantly reduced the rate of body weight gain in DHT rats and was not different from that of controls (Fig 2b).

### Abdominal adipose tissue

**Fig 3:**
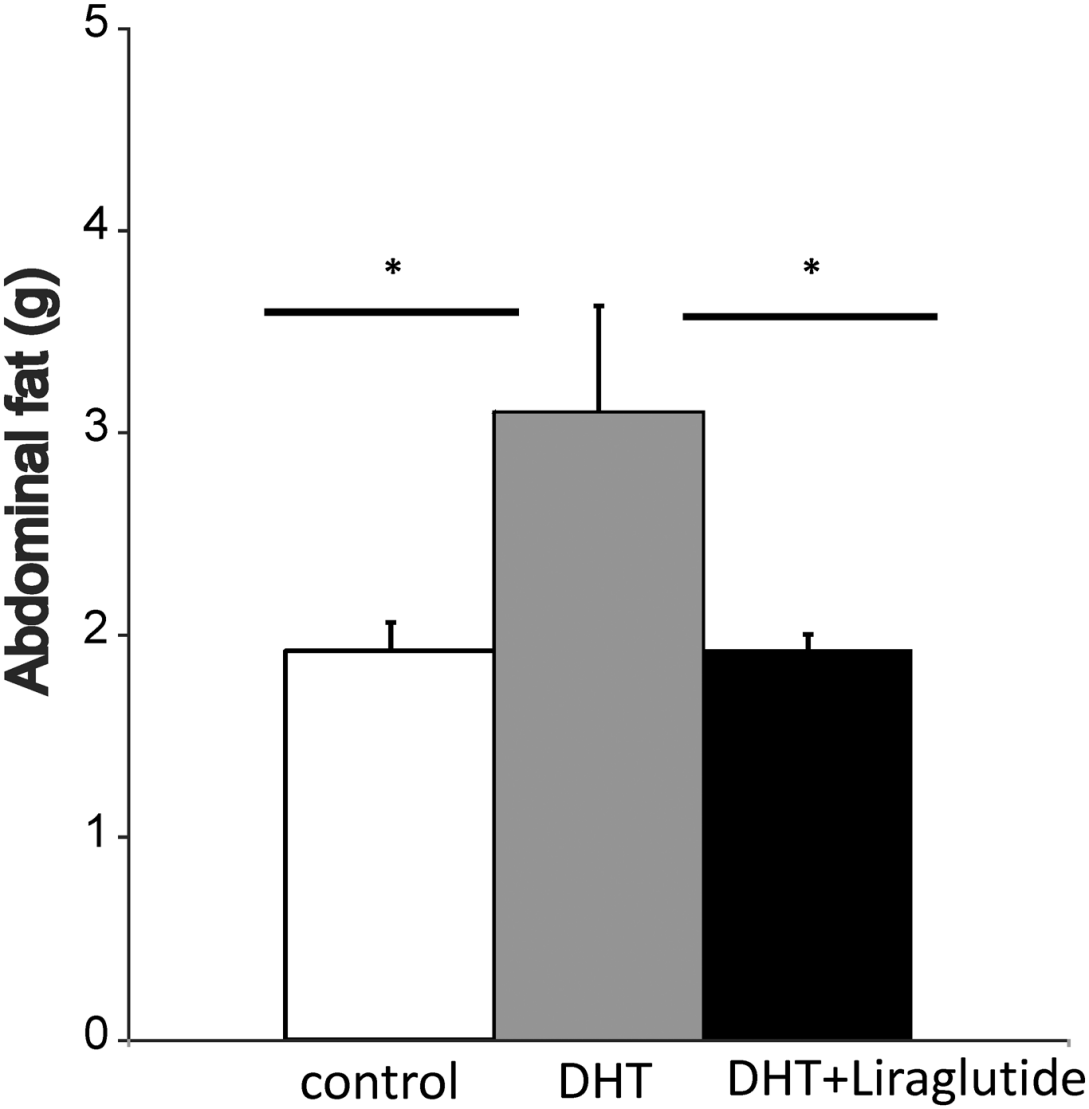
Effect of liraglutide on abdominal fat in DHT treated rats. (Mean± SEM; g): Abdominal fat was collected at end of study (88–90 days from pellet/sham implantation) in control (n = 8), DHT (n = 11) and DHT+Liraglutide (n = 8). * denotes p<0.05 between DHT + liraglutide and DHT and control.

Changes in adipose tissue mass in the abdomen in the three groups are shown in Fig 3, DHT treated rats had more abdominal adipose tissue at 88–90 days post DHT implantation compared to control rats (p = 0.04). Treatment with liraglutide for 4 weeks significantly reduced abdominal adipose tissue deposition (p = 0.02) and brought it down to control levels.

### Glucose sensitivity

**Fig 4:**
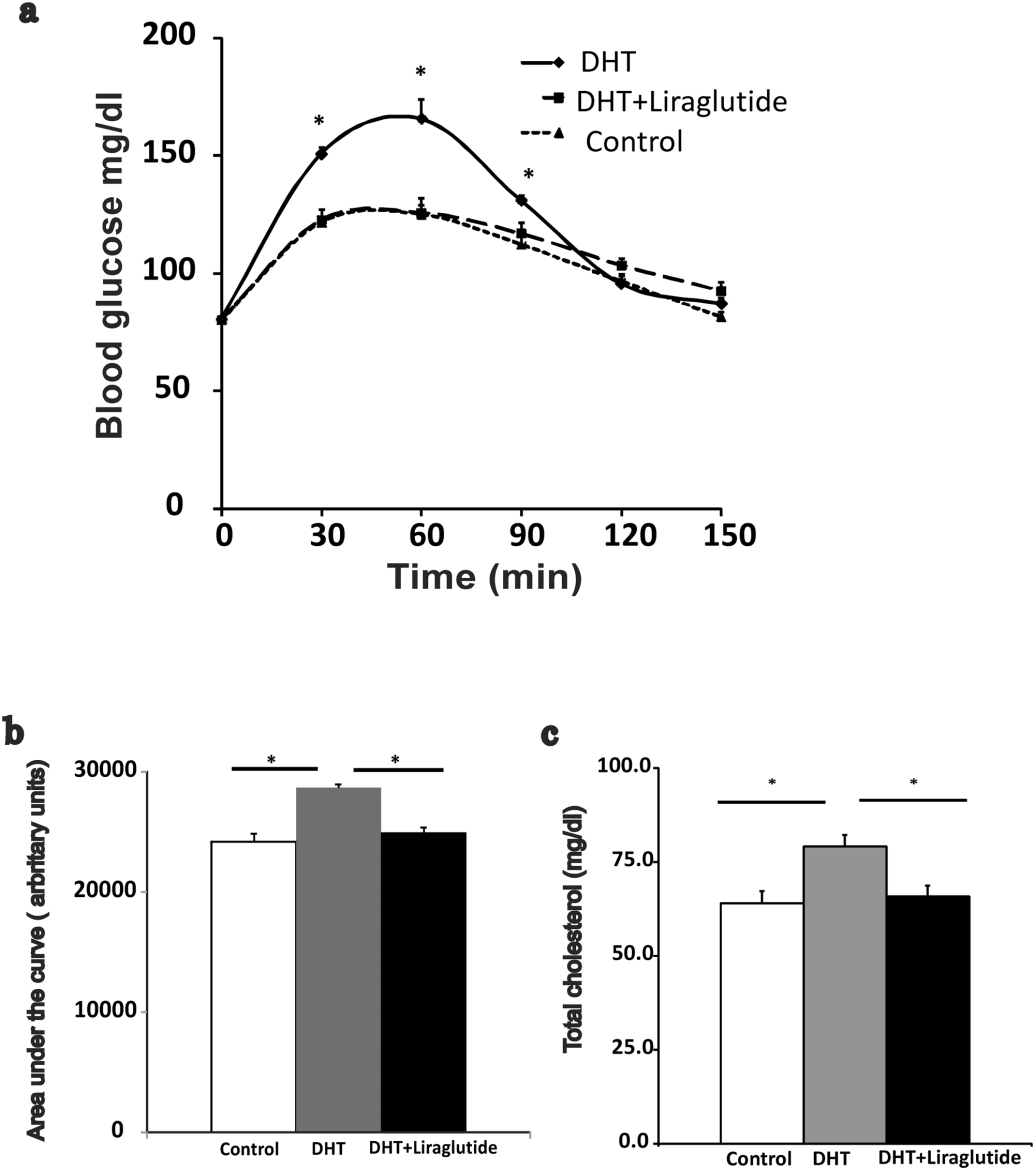
Effect of liraglutide on DHT induced metabolic perturbation. (Mean± SEM): a) oral glucose tolerance test done at 16 weeks (n = 9 control, n = 8 DHT+liraglutide and n = 10 DHT). b): Area under the curve during OGTT for different groups. c): Total cholesterol measured at the end of the study (88–90 days from pellet/sham implantation) after 10 hour fast (n = 8 rats in DHT and DHT+ liraglutide group and n = 5 in control group). * denotes p<0.05 between DHT and DHT+ liraglutide and DHT and control.

Fasting glucose levels were not different between the groups and neither were the fasting insulin levels (Control 5.5± 1.1 μIU/ml, DHT 8.2±1.5 μIU/ml, DHT+ liraglutide 5.8±1.1 μIU/ml). However blood glucose levels in DHT-treated animals were significantly higher than that in control animals post glucose load at 30, 60 and 90 minutes (p<0.01). On the other hand, treatment with liraglutide significantly reduced blood glucose levels at 30, 60 and 90 min post glucose load in the DHT-treated rats (p<0.01) (Fig 4a). Area under the curve (AUC) in the three groups is shown in Fig 4b. The AUC was increased in DHT-treated rats compared to control (p<0.01). This effect was totally reversed by treatment with liraglutide (p<0.01).

### Total Cholesterol

Fig 4c shows the total cholesterol levels in the three groups. Total cholesterol increased significantly in DHT-treated rats compared to control rats (p<0.01). In contrast, liraglutide treatment significantly reduced total cholesterol levels compared to DHT-treated animals (p<0.01) and this was comparable to what was seen in control animals.

### Telemetry

**Fig 5:**
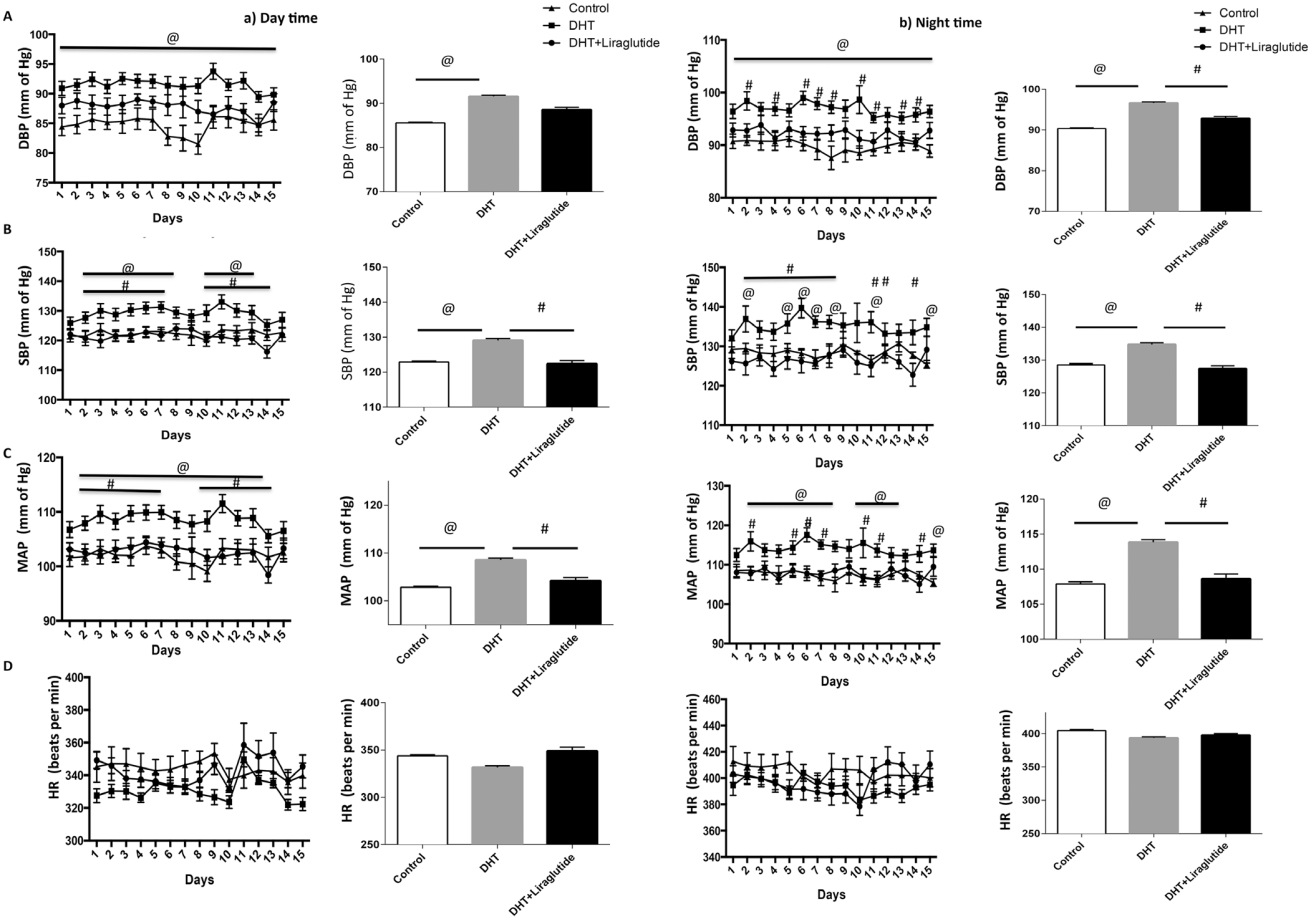
Effect of liraglutide on DHT induced perturbed blood pressure parameters. (Mean± SEM; mmHg)**:** a) Day time radiotelemeter data starting at 14 weeks of age: Data is averaged hourly over 15 days. Line graph depicts diastolic blood pressure (DBP; mmHg) (panel A), systolic blood pressure (SBP) (panel *B*), mean arterial pressure (MAP; mmHg) (panel *C*), and heart rate (HR; beats/min) (panel *D*), respectively b) Night time radiotelemeter data starting at 14 weeks of age: Data is averaged hourly over 15 days. Line graph depicts diastolic blood pressure (DBP; mmHg) (panel A), systolic blood pressure (SBP) (panel *B*), mean arterial pressure (MAP; mmHg) (panel *C*), heart rate (HR; beats/min) (panel *D*), respectively. Bar graphs depict average values of BP parameters shown in panels A-D. Significant difference (p<0.05) between groups is marked as follows: # = DHT vs DHT+ liraglutide @ = DHT vs control.

Changes in blood pressure in the three groups are shown in Fig 5. Day time blood pressures are depicted in Fig 5a and night time blood pressures in Fig 5b. Day time average DBP was significantly increased in DHT treated rats compared to control rats (p<0.05). Treatment with liraglutide produced modest reduction in average DBP in DHT treated rats that did not reach statistical significance (Panel A).

Average SBP during the entire observation period with DHT treatment was significantly elevated compared to control rats. However, treatment with liraglutide, significantly reduced SBP (p<0.05) (Panel B).

Mean arterial pressure (MAP) remained consistently high in DHT-treated rats compared to controls. Average MAP in DHT-treated animals was significantly higher compared to control rats (p<0.01). Liraglutide treatment produced a marked reduction in MAP in the DHT-treated rats (p<0.05) (Panel C). In contrast to blood pressure, there were no significant differences in heart rate between the 3 groups.

Blood pressure measurements during the night time were markedly higher in general compared to day time blood pressure (Fig 5b). Average DBP over the entire period of observation was significantly increased in DHT-treated rats compared to control rats (p<0.05). Treatment with liraglutide produced a marked reduction in average DBP in DHT-treated rats (p<0.05). Average systolic blood pressure (SBP) in DHT rats was significantly higher than the controls (p <0.05). However, treatment with liraglutide, significantly reduced SBP in DHT-treated rats (p<0.01). Average MAP were significantly higher in DHT-treated animals compared to control (p<0.01) and liraglutide treatment reduced the MAP in DHT-treated rats significantly (p<0.05).

## Discussion

In this study, we demonstrate for the first time that a GLP-1agonist has beneficial effects on metabolic perturbations, including weight gain, abdominal adiposity and systemic glucose homeostasis, and improves hypertension in a well-established hyperandrogenic rat model of PCOS.

PCOS is a condition that is characterized by the presence of polycystic ovaries, elevated circulating androgens with increased prevalence of obesity, insulin resistance and alterations in lipid profile[3, 26, 27]. More recently, younger women with PCOS have been reported to develop hypertension[28]. Although the exact mechanism leading to hypertension is not clear, gain in body weight and changes in lipid profile are possible contributing factors. Obesity can be present in 30–70% of women with PCOS[29] and obesity is a known independent risk factor for hypertension [30]. Moreover, obesity also precipitates insulin resistance and other metabolic disorders. Therefore it is not surprising that women with PCOS have increased prevalence of insulin resistance, impaired glucose tolerance and type 2 diabetes [11, 31]. Current treatment strategies target either the metabolic component or the hyperandrogenism and ovulation failure in PCOS. There is an urgent need for novel therapies that address the metabolic, cardiovascular and hormonal dysfunction in this syndrome.

Liraglutide is a long acting Glucagon-like peptide 1 (GLP-1) receptor agonist. It is FDA approved for treating type 2 diabetes and improves glycemic control by augmenting insulin secretion from the pancreas [32]. Liraglutide has also been shown to lower blood pressure in patients with type 2 diabetes [33]. Therefore, we hypothesized that liraglutide may be beneficial in simultaneously treating the metabolic and cardiovascular dysfunctions associated with PCOS. We found that liraglutide treatment produced a significant decrease in body weight, and significantly lowered abdominal adiposity in animals with PCOS in the present study. In humans, liraglutide promotes weight loss primarily by suppressing appetite and delaying gastric emptying[34–36]. A recent study also found that liraglutide treatment decreased the emotional eating score and uncontrolled eating score in PCOS women[37]. Similar mechanisms could be in place in the DHT-rat model of PCOS used in the present study.

PCOS is also frequently associated with type 2 diabetes and impaired glucose intolerance [31, 38]. It is seen not only in adult PCOS patients, but also in adolescent PCOS subjects. Higher levels of circulating blood glucose levels in these patients have been attributed to insulin resistance. These metabolic perturbations are frequently observed before the clinical diagnosis of PCOS in adolescents, especially when they are related to women with PCOS [39]. In some PCOS patients, fasting glucose levels can be impaired but others can have a normal fasting blood level with impaired glucose tolerance test[31]. It has been shown that in women with PCOS fasting plasma glucose measurements failed to diagnose 58% of women with diabetes that was diagnosed by a 2 hour OGTT[38]. In the present study, DHT-treated rats had normal fasting blood glucose level but had higher glucose levels during the OGTT compared to controls, similar to what is seen in PCOS women. Moreover, liraglutide treatment normalized glucose excursion in DHT-treated rats during the OGTT indicating that a tighter glycemic control was achieved. This suggests that liraglutide could potentially have beneficial effects in controlling blood glucose levels in PCOS patients.

In the present study, we also observed increases in abdominal adipose tissue deposition and higher levels of serum cholesterol in DHT-treated rats supporting findings from previous studies[19, 23]. One interesting finding in the present study was that liraglutide treatment was able to reduce abdominal obesity and serum cholesterol levels in DHT treated PCOS rats. In fact, the beneficial effect of liraglutide on lipid levels has been reported in another animal model of metabolic syndrome[40] and supports our findings. Recent human studies indicate that liraglutide in combination with metformin and lifestyle interventions can indeed decrease obesity in PCOS women[41]. Liraglutide treatment was also found to improve satiety, decrease feeding and lead to weight loss in PCOS women[37]. This indicates that liraglutide could affect feeding circuits in the brain [42]. Further studies are needed to investigate this possibility.

PCOS patients have higher prevalence of hypertension and this is being reported in much younger patients in recent years[11, 43]. Moreover, hyperandrogenic PCOS patients are reported to have a higher incidence of hypertension compared to non-androgenic PCOS patients [44].

Hyperandrogenism is also an independent risk factor for metabolic syndrome in adolescents. One study reported that 37% of adolescents with PCOS had metabolic syndrome after adjusting for body mass index and insulin resistance. In the same study prevalence of hypertension was greater in girls with PCOS compared to controls[8]. The mechanism by which hypertension develops in PCOS patients is not clear. In the DHT induced rat model of PCOS increased arterial wall rigidity, and enlarged vascular lumen are believed to contribute to the elevation in blood pressure observed [45]. Other causes of hypertension could be increased vascular endothelial dysfunction and elevated vascular prostanoid production[20], reduced nitric oxide bioavailability and increased production of reactive oxygen species[46–49]. The precise mechanism by which GLP-1 analogs lower blood pressure is not clear but they could act at multiple sites to produce this effect[50]. Based on our findings, it is likely that the improved systemic glucose homeostasis, marked reduction in body weight and abdominal obesity with liraglutide treatment could also result in reduction of hypertension in the DHT induced rat model of PCOS. Future studies will be addressing the mechanism by which GLP-1 agonist improves the cardiometabolic abnormalities seen in this model.

Diet and exercise have been shown to have a positive impact on menstrual irregularity, hirsuitism and insulin resistance in PCOS [51] but this for most people, is not a sustainable routine. Therefore, there is a need to identify diverse therapeutic strategies that can ameliorate the multiple deleterious effects observed in PCOS. Results from our study indicate that liraglutide can indeed improve metabolic perturbations and hypertension in this rat model of PCOS. GLP-1 agonists that have beneficial effects on glycemic control, weight gain and blood pressure in type 2 diabetes should continue to be considered for clinical trials in PCOS to assess their ability to chronically improve the multiple manifestations of the syndrome. Although liraglutide treatment decreased glucose excursion during OGTT, total cholesterol, weight gain and above all hypertension over a short period of 4 weeks, we did not observe improvements in estrous cyclicity. It is possible that chronic use of liraglutide may lead to improvement in this aspect also. Further studies addressing the exact mechanism by which GLP-1 agonist alleviates the cardiometabolic perturbations in PCOS are required to develop new therapeutic agents targeting the GLP-1 system in this condition.

## Conclusion

With the increasing prevalence of polycystic ovarian syndrome in adolescent females and higher incidence of hypertension in this syndrome, it is vital to study and target this aspect of the syndrome to prevent long term cardiovascular morbidity. Hence, development and or discovery of therapeutic agents that address not only the hyperandrogenism and insulin resistance observed in PCOS but also the cardiovascular impairment seen in this condition are crucial. Our study provides early evidence of the beneficial effects of a GLP-1 agonist in improving glucose sensitivity, abdominal obesity, serum cholesterol and hypertension. Further studies are required to specifically understand the role of GLP-1 in cardiovascular impairment in PCOS to develop new therapeutic strategies.
